# Synthesis, antibacterial, antibiofilm, and docking studies of chalcones against multidrug resistance pathogens

**DOI:** 10.1016/j.heliyon.2024.e30618

**Published:** 2024-05-06

**Authors:** Tariq Nawaz, Affifa Tajammal, Aisha Waheed Qurashi, Mehr-un Nisa, Dalal Nasser Binjawhar, Munawar Iqbal

**Affiliations:** a Department of Chemistry, Lahore Garrison University, Pakistan; b Department of Biology, Lahore Garrison University, Pakistan; c Department of Chemistry, The University of Lahore, 1-km Defence Road, Lahore, Pakistan; d Department of Chemistry, College of Science, Princess Nourah bint Abdulrahman University, P.O. Box 84428, Riyadh, 11671, Saudi Arabia; e School of Chemistry, University of the Punjab, Lahore 54590, Pakistan

**Keywords:** Antibacterial activity, Antibiofilm activity, Chalcones, GlcN-6-P synthase inhibition

## Abstract

The escalating threat of drug-resistant microbes underscores the urgent need for novel antimicrobial agents. In response, considerable research effort has been directed towards developing innovative frameworks and strategies to address this challenge. Chalcones, known for their broad-spectrum biological activities, have emerged as promising candidates for combating drug resistance. In this study, a series of 2′-Hydroxychalcones (**5a, 5b, 5c,** and **5d**) with varying electron withdrawing and donating groups were synthesized via Claisen Schmidt condensation. FT-IR, ^1^H NMR, and ^13^C NMR analyses were employed to confirm the structure of the synthesized compounds. Subsequent evaluation of the synthesized compounds revealed their potential as antibacterial and antibiofilm agents. Notably, compounds **5a** and **5d** exhibited potent antibacterial activity against multidrug-resistant (MDR) bacteria *E. coli*, *P. aeruginosa, K. pneumoniae, and S. aureus,* surpassing the reference drug Ciprofloxacin (30 μg/mL) and other synthesized compounds. Compound 5d showed a notable 19.5 mm zone of inhibition against *K. pneumoniae*. Furthermore, **5a** (at a concentration of 30 μg) and **5d** (at a concentration of 50 μg) exhibited statistically significant (P > 0.05) biofilm inhibition efficacy compared to Ciprofloxacin (30 μg/mL). The synthesized chalcones **5a-5d** were also docked via *PachDock* molecular docking software for Glucosamine-6-phosphate (GlcN-6-P) synthase inhibition and showed that ligand **5a** exhibited outstanding results with score 4238 and ACE value −160.89 kcal/mol, consistent with the observed antibacterial activity. These findings underscore the potential of chalcones, particularly **5a** and **5d**, as promising candidates for the development of new antimicrobial agents targeting drug-resistant microbes and biofilm formation.

## 1 Introduction

The perpetual battle against infectious diseases has been ongoing for humanity. In modern times, the treatment of these diseases necessitates the use of a multi-drug regimen for an extended duration. Multidrug resistance phenomenon has resulted in increased mortality, morbidity and higher healthcare costs. Unfortunately, this approach has resulted in the swift emergence of strains that are resistant to multiple drugs, as well as a significant problem of patient non-compliance [1]. Over the past sixty years, antibiotics played a prominent role for the treatment of toxins produced by bacteria. In recent years, there is a huge rise in the range of bacteria that are harmful and resistant to antibiotics [2]. The huge problem now days are multidrug resistance bacteria such as Vancomycin-Resistant *Enterococci* etc which cause numerus infections [3]. The increased MDR bacterial response towards antibiotics ultimately results in more drastic situations in terms of difficult-to-treat biofilm-related infections. Biofilms are intricate, multicellular communities of bacteria within an extracellular matrix. These structures have been shown to be associated with a significant proportion, approximately 80 %, of all bacterial infections [4]. Biofilm formation has been shown to promote a significant increase in antibiotic tolerance, reaching levels up to 1000 times higher than what is typically observed in planktonic bacteria (individual, free-floating bacteria). Additionally, the formation of biofilms has been connected to long-lasting infections in situations like lung pneumonia in individuals with cystic fibrosis, otitis media, chronic wounds that don't heal, and the contamination of medical implants. This association with biofilms can lead to ineffective treatment of these infections, posing a considerable challenge to healthcare interventions. In order to provide new effective therapeutic agents, vigorous efforts are therefore required, and the development of new synthetics for microbial targets is a major problem for medical chemistry [5]. General clinical experience indicates that single target drugs, although having good inhibitory activity against a specific target, are not effective agents for the biological system [6]. This methodology creates it impossible to define a unique cure and specifically or at certain stages of its synchronization targets the virion without destroying the host cells [7].

**Fig. 1:**
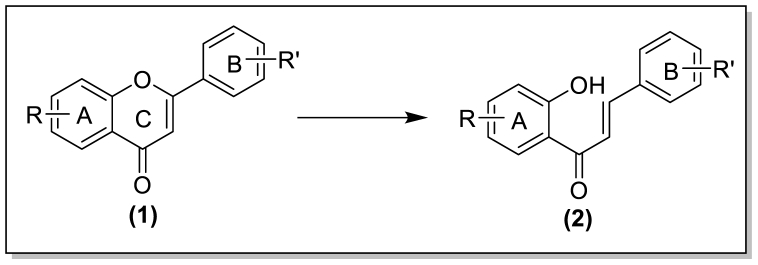
Conversion of flavone to chalcone.

Chalcone scaffold is an open-chain flavonoids obtained from C-ring cleavage in flavonoids [8] (Fig. 1), serves as a fundamental structure that demonstrates diverse activities such as antimalarial [9], anticancer [10], anti-inflammatory [11], antiprotozoal [12], *anti*-HIV [13], antioxidant [14], antiulcer [15], antileishmanial [16], antitumor [17], antiparasitic [18], antihyperglycemic [19], antiproliferati [20] and antiAlzheimer's [21] with a particular emphasis on its antimicrobial properties [22].

Certain substituted chalcones and their derivatives, along with some of their heterocyclic analogues, have been documented for their potent biological properties, which have been shown to be harmful to the growth of microbes, tubercle bacilli, malarial parasites, and intestinal worms. Furthermore, it offers a versatile foundation for the construction of heterocyclic scaffolds through combinatorial assembly during synthesis [23].

**Fig. 2:**
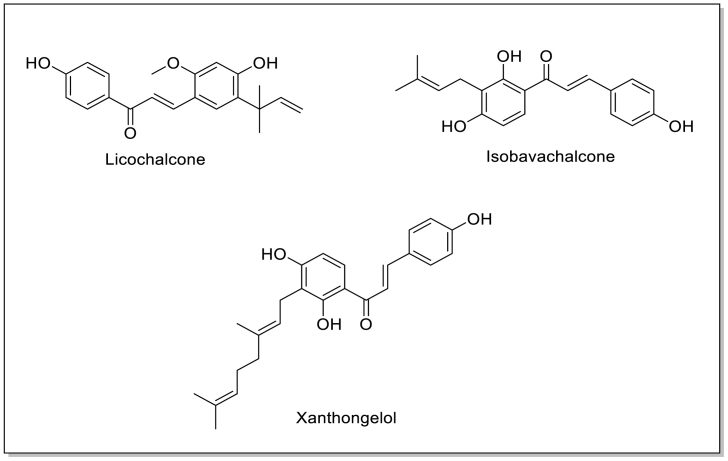
Structure of some naturally occurring Chalcones.

Many chalcones such as Licochalcone, Xanthongelol, Isobavachalcone and other chalcones derivative (Fig. 2) have been approved for clinical use which shown an excellent antimicrobial activity [24]. Since the chalcone spine is very useful in the preparation of substances with medicinal potential for different diseases [25] Several chalcone derivative like Isobavachalcone was extracted from *Psoralea corylifolia* which demonstrated significant antimicrobial effect with MIC of 8 μg/mL on *MRSA* strains [26]. Chalcone exhibited significant antimicrobial activity against both *Staphylococcus aureus* and *Escherichia coli* [27]. In 2023, M. Yadav et al. synthesized a chalcone derivative which showed MIC of 0.012 mol/mL against *P. aeruginosa*. Furthermore, a molecular docking study indicated that the chalcones have the potential to act as antibacterial inhibitors [28]. A. Asadzadeh et al. in 2023, synthesized chalcone derivatives showed a good binding affinity to Streptococcus mutans Sortase A [29].

Glucosamine-6-phosphate synthase (G-6-P synthase) is a pivotal enzyme for the synthesis of UDP-N-acetyl glucosamine. G-6-P synthase is crucial for the initial steps in hexosamine biosynthesis. One of the products catalyzed by this enzyme, N-acetyl glucosamine, holds significant importance in forming the peptidoglycan layer present in the cell walls of both bacteria and fungi. G-6-P synthase holds great potential as a promising target for the discovery of novel antimicrobial compounds. Evaluating these potential compounds for their preservative efficacy may lead to the identification of safer and more effective preservatives for various applications [30]. Chalcones are proved to be potential GlcN-6-P synthase inhibitors [31,32]. Taking into consideration the therapeutic significance of chalcones and as part of our ongoing research efforts to develop biologically potent molecules [33,34]. Present research work is based on hypothesis that tested compounds might have promising antibacterial activities as novel compound against MDR bacteria. This could potentially help mitigate healthcare challenges and offer promising prospects for effective treatment.

## 2 Experimental

### 2.1 Chemistry

We monitored the advancement of the reaction by employing silica gel 60 F254 TLC plates and visualizing the spots under UV radiation. FT-IR spectra were recorded using Agilent Technologies 41,630. Additionally, ^1^H NMR and ^13^C NMR spectra were obtained using AVANCE AV-400 MHz and AVANCE AV-500 MHz, Bruker 125 MHz, respectively.

### 2.2 General method for the synthesis of 2-hydroxy-5-nitro chalcones (5a-5d)

In a stirred solution of 2-Hydroxy-5-nitroacetophenone (6 mmol) and aromatic benzaldehyde (6 mmol) in 25 mL of ethanol, KOH (20 % w/v aqueous solution, 6 mL) was added. The mixture was stirred at room temperature for 24–36 h. The progress of the reaction was checked using TLC with a hexanes:ethyl acetate (7:3) solvent system. Afterward, the reaction mixture was cooled on ice and acidified with HCl (10 % v/v aqueous solution). The resulting product was recrystallized using ethanol to obtain compounds (**5a, 5b, 5c, 5d**) [35].

### 2.3 (*E*)-3-(4-Fluorophenyl)-1-(2-hydroxy-5-nitrophenyl)prop-2-en-1-one (5a)

Yield: 1.2 g, 70 %, Appearance: yellow needles, m. p: 230–233 °C, Rf 0.65, FT-IR: ʋ (cm^−1^) 3267 (-OH), 3098 (=C–H), 1642 (C=O), 1506, 1473 (C=C), 1565, 1348 (-NO_2_), 1192 (C–F). ^1^H NMR: (500 MHz/DMSO‑*d*_6_): *δ* 12.76 (1H, s, 2ʹ-OH), 8.76 (1H, s, 6ʹ-H), 8.36 (1H, d, *J* = 8.8 Hz, 4ʹ-H), 7.98 (2H, bs, 2-H, 6-H), 7.87–7.78 (2H, m, *α-H, β-H*), 7.35–7.31 (2H, m, 3-H, 5-H), 7.21 (1H, d, *J* = 9.0 Hz, 3ʹ-H). ^13^C NMR: (125 MHz/DMSO‑*d*_6_): δ 191.96 (C=O), 165.27(2ʹ-C), 163.18 (4-C), 144.52 (β-C), 132.11 (^m^*J*_C-F_ = 8.8 Hz, 2-C, 6-C), 131.52 (^p^*J*_C-F_ = 2.5 Hz, 1-C), 130.19 (4ʹ-C), 127.09 (6ʹ-C), 123.86 (1ʹ-C), 123.67 (3ʹ-C), 118.99 (α-C), 116.56 (^o^*J*_C-F_ = 21.6 Hz, 3-C, 5-C).

### 2.4 (*E*)-3-(4-Chlorophenyl)-1-(2-Hydroxy-5-nitrophenyl) prop-2-en-1-one (5b)

Yield: 1.3 g, 72 %, Appearance: Yellow needles, m. p: 231 °C, Rf 0.71, FT-IR: ʋ (cm−1): 3257 (-OH), 3123 (=C–H), 1649 (C=O), 1523, 1464 (C=C), 1565, 1363 (-NO2), 635 (C–Cl). ^1^H NMR: (500 MHz/DMSO–d6): *δ* 12.75 (1H, s, 2ʹ-OH), 8.74 (1H, s, 6ʹ-H), 8.35 (1H, d, *J* = 9.0 Hz, 4ʹ-H), 7.92–7.89 (3H, m, *α-H*, 2-H, 6-H), 7.77 (1H, d, *J* = 15.6 Hz, *β-H*), 7.55–7.53 (2H, m, 3-H, 5-H), 7.19(1H, d, *J* = 9.0 Hz, 3ʹ-H). ^13^C NMR: (125 MHz/DMSO–d_6_): δ 191.83 (C=O), 165.26 (2ʹ-C), 144.09 (β-C),140.10 (5ʹ-C), 136.07 (4- C), 133.78 (1-C), 131.30 (2-C, 6-C), 130.20 (4ʹ-C), 129.53 (3-C, 5-C), 127.14 (6ʹ-C), 124.71 (1ʹ-C), 123.64 (3ʹ-C), 119.00 (α-C).

### 2.5 (*E*)-3-(4-Bromophenyl)-1-(2-hydroxy-5-nitrophenyl)prop-2-en-1-one (5c)

Yield: 1.5 g, 71 %, appearance: yellow needles, m. p: 232–235 °C, Rf 0.75, FT-IR: ʋ (cm^−1^) 3302 (-OH), 3092 (=C–H), 1673 (C=O), 1562, 1432 (C=C), 1535, 1356 (-NO_2_), 554 (C–Br). ^1^H NMR: (500 MHz/DMSO‑*d*_6_): *δ* 12.71 (1H, s, 2ʹ-OH), 8.74 (1H, s, 6ʹ-H), 8.36 (1H, d, *J* = 9.0 Hz, 4ʹ-H), 7.91 (1H, d, *J* = 15.6 Hz, *α-H*), 7.85 (2H, d, *J* = 7.8 Hz, 2-H, 6-H), 7.76 (1H, d, *J* = 15.6 Hz, *β-H*), 7.69 (2H, d, *J* = 7.9 Hz, 3-H, 5-H), 7.20(1H, d, *J* = 9.1 Hz, 3ʹ-H). ^13^C NMR: (125 MHz/DMSO‑*d*_6_): δ 191.19 (C=O), 165.19 (2ʹ-C), 144.16 (β-C), 140.07 (5ʹ-C), 134.13 (1-C), 132.48 (3-C, 5-C), 131.49 (2-C, 6-C), 130.19 (4ʹ-C), 127.14 (6ʹ-C), 124.99 (1ʹ-C), 124.88 (3ʹ-C), 123.78 (4-C), 119.00 (α –C).

### 2.6 (*E*)-1-(2-Hydroxy-5-nitrophenyl)-3-(4-methoxyphenyl)prop-2-en-1-one (5d)

Yield: 1.1 g, 61 %, Appearance: yellow needles, m. p: 201–203 °C, Rf 0.60, FT-IR: ʋ (cm^−1^) 3570 (-OH), 3081 (=C–H), 1635 (C=O), 1558, 1472 (C=C), 1550, 1341 (-NO_2_). ^1^H NMR: (500 MHz/DMSO‑*d*_6_): *δ* 12.95 (1H, s, 2ʹ-OH), 8.79 (1H, s, 6ʹ-H), 8.36 (1H, d, *J* = 9.1 Hz, 4ʹ-H), 7.88 (2H, d, *J* = 7.9 Hz, 2-H, 6-H), 7.80 (2H, s, *α-H, β-H*), 7.19 (1H, d, *J* = 9.1 Hz, 3ʹ-H), 7.05 (2H, d, *J* = 7.9 Hz, 3-H, 5-H). ^13^C NMR: (125 MHz/DMSO‑*d*_6_): δ 192.08 (C=O), 165.54 (2ʹ-C), 162.38 (4-C), 146.26 (β-C), 140.08 (5ʹ-C), 131.84 (2-C, 6-C), 130.13 (4ʹ-C), 127.44 (1-C), 127.02 (6ʹ-C), 123.39 (1ʹ-C), 120.99 (3ʹ-C), 119.02 (α –C), 115.03 (3-C, 5-C), 55.94 (OCH_3_).

### 2.7 *In vitro* antibacterial activity

The synthesized compounds (5a-5d) were assessed for their antibacterial activities using the disc-diffusion method on Muller Hinton agar medium [36]. This study utilized Multidrug resistant (MDR) bacteria; *E. coli, P. aeruginosa, K. pneumoniae,* and *S. aureus*) bacteria previously isolated from cancer patients at a tertiary care hospital in Lahore, Pakistan. For the preparation of stock solution of each test compound, 10 mg of the compound was dissolved in 10 mL of DMSO. Following this, different concentrations, ranging from 30, 50, 100, 200, 400, to 800 μg/mL were prepared by diluting the stock solution accordingly. The paper disks (5.0 mm) were soaked in each concentrations for 5 min were placed on the surface of the freshly swabbed bacterial cultures on swabbed nutrient agar plates. The antibiotic disk of Ciprofloxacin (30 μg/mL) was used as standard drug and negative control at same plate. Following this, the plates were placed in an incubator set at 37 °C for a duration of 24 h. The zones of bacterial growth inhibition by each compound were then measured in millimeters (mm).

### 2.8 Antibiofilm assay (microreader plate)

To assess the efficacy of synthesized chalcones in inhibiting biofilm formation by bacterial pathogens (*E. coli, P. aeruginosa, K. pneumoniae, and S. aureus*), we quantitatively analyzed biofilm formation using a microplate reader, following the method outlined by Qurashi & Sabri [37]. Microreader plate is used to quantify biofilm formation. Microbial culture is prepared in Luria-Bertani (LB) Broth and adjusted to 0.5 at OD-600 nm to ensure equal cell density 10^8^ CFU/mL 180 μl of bacterial culture was dispensed into each well of sterile polystyrene 96-well-flat bottom plates. Then, 20 μl of each of the eight chalcone derivatives with varying concentrations were added using DMSO as a solvent. (30, 50 μg/mL). The microplates were placed in the incubator at 37 °C for a duration of 92 h, a time previously determined to be optimal for these cultures, without agitation. Afterward absorbance of planktonic cells was recorded at 600 nm. Next, the liquid culture was removed, and each well was stained with 100 μL of 1 % aqueous crystal violet for 20 min at room temperature. After 20 min of staining of adhered cells alongside walls of plates, rinsing with sterile distilled water was done. After drying, 200 μL of 33 % acetic acid was added to each well to dissolve the adhered cells. Following this, the absorbance of the plates was measured at 570 nm using a microplate reader. The Biofilm pattern of adhered cells was quantified as Normalized value (OD 570 nm/OD 600 nm) by excluding the difference of planktonic cells. All assays were carried out in triplicates. Classification of bacterial adherence summarized in Graph, based on “ANOVA” with Fisher's least significant difference (LSD) values.

## 3 Results and discussion

**Scheme 1:**
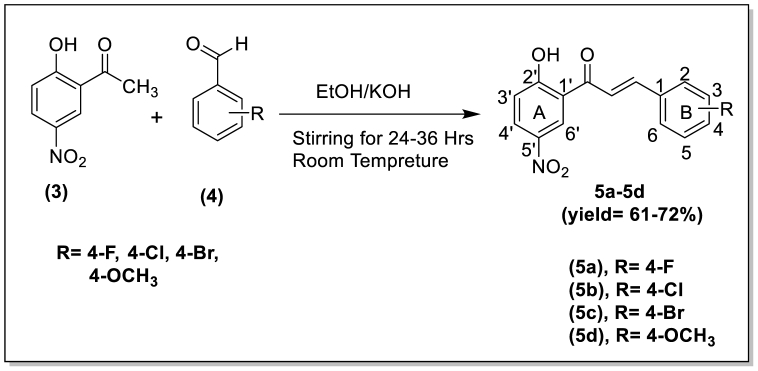
Scheme for the synthesis of 2-hydroxy-5-nitro chalcone.

**Fig. 3:**
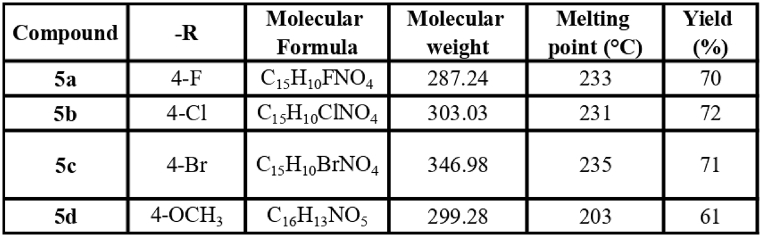
Physicochemical characterization data of the synthesized compounds.

Chalcones were synthesized through Claisen-Schmidt condensation reaction, This involved reacting 2-Hydroxy-5-nitroacetophenone (1) with different aromatic aldehydes in the presence of KOH (Scheme 1). The synthesis of chalcones resulted in satisfactory yields, ranging from 58 % to 72 %. To characterize the synthesized compounds, infrared (IR), ^1^H NMR, and ^13^C NMR techniques were employed. In the ^1^H NMR data of compounds **5a-5d**, the presence of 2ʹ-OH was confirmed by observing signals in the range of δ12.71 - δ12.95. Signals within the range of δ7.75 – δ7.93 indicated the presence of *α-H* and *β-H*. Notably, the observed coupling constant value of 15 Hz clearly pointed to the trans configuration of the chalcones. Furthermore, other aromatic protons were also detected in their respective regions during the analysis. Fig. 3 presents a summary of the physiochemical data of the synthesized compounds.

### 3.1 Antibacterial activity

Chalcones are known for their wide range of biological activities, which are affected by the substitutions attached to the chalcone ring. In this study, the antibiotic potential of the synthesized compounds was assessed individually using the Kirby Bauer disk diffusion method. The results were quantified by measuring the zone of inhibition (ZOI) for each compound, alongside positive and negative controls. Chalcone derivatives that displayed a ZOI greater than 6 mm were considered to have promising antimicrobial properties [39]. Among the chalcone derivatives tested (**5a-5d**), all demonstrated *in vitro* antimicrobial activity against a panel of standard strains of Gram-positive bacteria (*S. aureus*) and Gram-negative bacteria (*E. coli, P. aeruginosa*, and *K. pneumoniae*). Compounds **5a** and **5d** demonstrated the most significant antibacterial efficacy against the examined pathogens at a concentration of 50 μg/mL, exceeding both the reference drug Ciprofloxacin (30 μg/mL) and all other synthesized compounds. There was a general trend of higher growth inhibition in terms of zone development by isolates *P. aeruginosa* and *S. aureus* as compared to rest of bacterial isolates. These findings highlight the potential of compounds **5a** and **5d** as potent antibacterial agents. Compound 5a features an electron-donating –OH group at position 2 and an electron-withdrawing –NO_2_ group at position 5 of the A ring. Additionally, on B ring, compound 5a features an electron-withdrawing fluoro group at position 4. Compound **5a** (50 μ**g**/mL) has highest antimicrobial efficacy against tested pathogens with 21 mm zone of inhibition (ZOI) against *E. Coli,* 28 mm against *P. aeruginosa,* 29 mm against *S. Aureus and* 21 mm against *K. pneumoniae* which is comparable to standard ciprofloxacin (Table 1). It was reported that chalcones containing 2,4-dichlorophenyl and 2,4-difluorophenyl rings proved to be potent antibacterial agents [38]. Presence of fluoro group on either A or B ring of chalcones activate the compound for antibacterial activity [39,40]. Lagu et al. have reported that the chalcones bearing trifluoromethoxy group have more effective antibacterial activity [41]. Compound **5d** has electron donating –OH group at position 2 and electron withdrawing –NO_2_ group at position 5 of A ring. Additionally, B ring 5d has electron donating methoxy group at position 4, Compound **5d** showed excellent antimicrobial efficacy with 22 mm zone of inhibition (ZOI) against *E. Coli,* 29 mm against *P. aeruginosa,* 28 mm against *S. Aureus and* 19.5 mm against *K. pneumoniae* which is comparable to standard ciprofloxacin (Table 1). So the compound **5a** and **5d** has showed comparable zone of inhibition as compared to other synthesized compounds as well as standard as shown in Table 1. Shaikh et al. have reported that the chalcones bearing methyl groups are significant antibacterial agents [42].

**Table 1:** Antimicrobial activity of compounds **(5a-5d)**.

Sr. No.	Conc.	*E.* *Coli*	*P. aeruginosa*	*S.* *Aureus*	*K. pneumoniae*	Substituent Properties
	μg/mL	Diameter of zone of inhibition in (mm)				
**5a**	**30**	15	16	12	13	**A Ring:** Electron-donating and withdrawing group **B ring:** Electron withdrawing
	**50**	21	28	29	21	
**5b**	**30**	_	_	_	**_**	**A Ring:** Electron-donating and withdrawing group **B ring:** Electron withdrawing
	**50**	16	17	23	12	
**5c**	**30**	_	**_**	_	_	**A Ring:** Electron-donating and withdrawing group **B ring:** Electron withdrawing
	**50**	_	_	_	_	
**5d**	**30**	13	14	10	12	**A Ring:** Electron-donating and withdrawing group **B ring:** Electron donating group
	**50**	22	29	28	19.5	
**Cipro.**	**30**	21	28	28	20	

This information is supported by Konduru et al. (2013) who observed antibacterial activity of hybrids Halo-chalcone (F, Cl, Br) which showed significant efficacy against *B. subtilis* [43]. The significant antimicrobial activity of the chalcone molecule might be due to the methoxy and hydroxyl groups present on ring A or ring B [44]. These functional groups likely contribute to the molecule's ability to effectively combat microbial pathogens [45,46]. In general, chalcone with groups (-OH and methoxy) statistically enhanced the antibacterial activity [47]. The lipophilicity of ring A in the hydroxyl chalcones was significant for their antibacterial activity [48]. At MIC 6.25 g/mL against the tested microorganisms, hydoxy and haloginated chalcone compound shown good antibacterial activity [49]. Li et al. have shown that chalcones bearing hydroxyl groups are active antibacterial agents against gram positive bacteria [50]. Syahri, J. et al. synthesized 2-hydroxy linked chalcones that exhibits significant antibacterial efficacy against different gram positive and gram negative bacteria [51]. Cationic groups such as amino group on A or B ring of chalcones are responsible for antibacterial activity [52].

### 3.2 Antibiofilm activity

Biofilm is a type of bacterial association usually developed on abiotic and biotic surfaces or at interfaces between air and liquid, covered by a extracellular bacterial polysaccharides, different proteins and nucleic acids in which bacteria multiply. Microbial biofilm-related infections are an essential part of both symptomatic and chronic bacterial diseases in both humans and animals and also cause food spoilage [53]. Biofilm colonies offer significant advantages to bacteria in terms of protective creation, but they are notorious for their antibiotic resistance. Chronic biofouling, food degradation, water pollution, and toxic substances are all caused by bacterial biofilms [54]. Most antimicrobials drugs are unable to penetrate the biofilm because to Efflux pump (EP), which acts as a secondary barrier to bacterial cells. It is the main reason of antibiotic resistance through combinatorial mechanism of drug and efflux pump inhibitors the antibiotic resistance could be reduced. Chalcones have been tested for EPI activity against *S. aureus* native pump NorA As a result, in biofilms chalcone have been found to be effective in degrading Efflux pump (EP) [55]. Quorum sensing (QS) system plays a crucial role in coordinating the formation of biofilms, secretion of virulence factors, mobility, and development of drug resistance, thereby influencing crucial bacterial cellular processes. Chalcones are proved to be active agents against quorum sensing to lower down the biofilm formation [56].

Biofilm formation of bacterial pathogens was tested in the presence and absence of synthesized compounds. The current study demonstrates that chalcones is effective against harmful bacteria that form biofilms. The capacity of chalcones to remove biofilms and disperse them makes it particularly well suited to the treatment of invasive bacteria and illnesses [57].

*Anti*-biofilm activity of chalcone against pathogenic isolates showed consistent results. So the efficacy of the four synthesized chalcones on the inhibition of the biofilm shows significant results (P > 0.05) for pathogens. The highest biofilm formation was recorded by *S. aureus* and *K. pneumoniae* as compared to the rest of isolate treatment and controlled experiment in (Fig. 5). However, in planktonic stage, the growth of *P. aeuroginosa* was highest as compared to all other pathogens but the trend was reversed for adherence (Normalized biofilm).

**Fig. 4:**
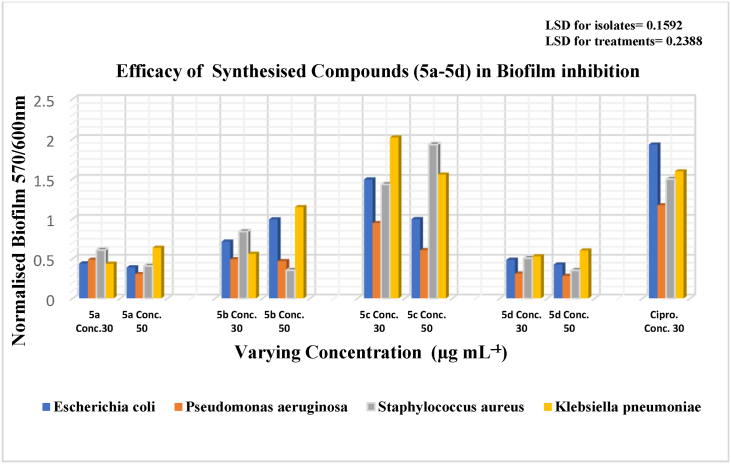
Antibiofilm efficacy of **(5a-5d)** against pathogens.

**Fig. 5:**
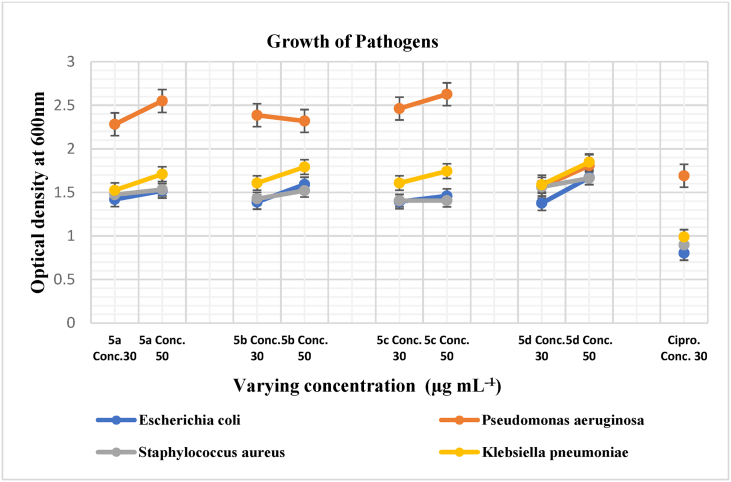
Effect of **(5a-5d)** on growth of *E. coli, P. aeuroginosa, K. pneumoniae* and *S. aureus*.

In Fig. 4, the biofilm assay demonstrated that compounds **5a** (at a concentration of 30 μg) and **5d** (at a concentration of 50 μg) exhibited the most effective inhibition against all tested pathogens as compared to the reference drug Ciprofloxacin (used at 30 μg/mL) and other synthesized compounds. The chalcone derivative **5a**, featuring hydroxyl and nitro groups in the A ring and a fluorine group in the B ring, displayed the most potent biofilm inhibition against all tested pathogens. **5b** also with the hydroxyl and Nitro groups as A ring, but other side of ring chloro group as ring B, showed the good biofilm inhibitory activity against bacterial strains (*E. coli, P. aeuroginosa, K. pneumoniae* and *S. aureus*). **5c** showed antibiofilm activity against *P. aeuroginosa* due to hydroxyl group on ring B, but this compound not effective against other three bacteria. Compound **5d** demonstrated notable efficacy in inhibiting pathogens (*E. coli, P. aeruginosa, K. pneumoniae, and S. aureus*) and produced highly significant results (P > 0.05) regarding biofilm inhibition.

The findings presented align with the research conducted by Nijampatnam et al. in 2016, which also demonstrated that chalcone derivatives hold promise in hindering the formation of biofilms. The study supports the notion that these compounds can be effective in inhibiting biofilm development and validates the antimicrobial properties observed in this current investigation. Hydroxyl group and methoxy group of chalcones prevent the formation of *S. aureus* biofilm and Slobodníkova L. et al. assessed (2016) phloretin also reduced the effectiveness of *E. coli* biofilm without preventing the growth of microbes [58,59]. Liu, M. et al., also assessed that xanthohumol is prenylated prevent *S. aureus* permeability and biofilm formation. In an already established biofilm, it also inactivated bacteria, likely disrupting the cohesion of the bacterial cytoplasmic membrane [60]. Chalcones without hydroxyl groups on B ring are typically more effective against bacterial membranes. Moreover strongly hydroxylated lipophilic groups on A ring chalcone could be more disruptive to the framework of the membrane [61]. Satokata et al., 2022 developed few hydoxy-haloginated chalcone which showed good antibiofilm activities 95 % inhibited cell viability at doses between 0.78 and 100 g/mL [49].

So this information is showed that the hydroxyl and methoxy groups compound disrupting more biofilm. **5a, 5b** and **5d** significantly reduced the biofilm formation by all pathogens (Fig. 5).

### 3.3 Molecular docking studies

To explore the specific outcomes of the structure-activity relationship (SAR) at the GlcN-6-P synthase, molecular docking experiments were performed at the receptor site. The molecular docking tool “Patch Dock” was utilized to dock ligands **5a-5d** with the crystal structure of GlcN-6-P synthase (PDB ID: 1MOQ) from the Protein Data Bank (PDB), as it is known for generating good molecular shape complementarity based on shape complementarity principles [62,63]. Among the different docking solutions, “solution 1″ was chosen because it contained the most critical residues surrounding the binding pockets. The docked structures were then analyzed using Discovery Studio 4.5 Visualizer. In Table 2, the scores and atomic contact energies (ACE) of the docked conformations are presented, along with details of the hydrogen bonds and hydrophobic interactions formed by each ligand within the binding pocket of the receptor protein. The manuscript further discusses the relative interactions of the synthesized chalcones **5a-5d** and the reference standard Ciprofloxacin with GlcN-6-P synthase (PDB ID 1MOQ) protein.

**Table 2:** Docking of ligands (**5a-5d**) to GlcN-6-P synthase (PDB ID: 1MOQ) using *Patch Dock*.

Ligand	Score^a^	ACE (kcal/mol)^a^	Amino Acids Show Potential for			Pi-alkyl Interactions
			H-bonds		Hydrophobic Interactions	
			Interactions	Distance (Å)^b^		
**5a**	4238	−160.89	Arg^472^, Tyr^576^, Asp^474^, Asn^522^	2.48, 3.27, 2.39, 2.54	His^566^, Val^567^, Ala^572^, Glu^569^, Gln^475^, Gly^473^, Pro^521^, Ala^551^	Ala^520^, Arg^472^
**5b**	3790	−92.94	Val^567^, Val^567^	2.66, 3.29	Glu^475^, Asp^474^, Gly^473^, Leu^525^,Asn^522^, Pro^521^, Ala^551^, Asp^548^, Tyr^576^	Ala^520^, Ala^572^
**5c**	3896	−92.78	Glu^569^	2.73	Val^567^, Tyr^576^, Ala^572^, Gln^475^, Asp^474^, Gly^473^, Leu^525^, Asn^522^, Pro^521^, Ala^551^, Asp^548^, His^566^	Ala^520^, Arg^472^
**5d**	3958	−102.77	Glu^569^	2.87	Ala^551^, Pro^521^, Asn^522^, Leu^525^, Gly^473^, Asp^474^, Gln^475^, Ala^572^, Tyr^576^, Val^567^, His^566^	Ala^520^, Arg^472^
Ciprofloxacin	4424	−227.85	Ser^303,^ Ser^347^, Thr^352^, Thr^352^, Val^605^	2.28, 2.18, 1.77, 2.91, 3.35	Gln^408^, Gln^348^, Leu^346^, Glu^396^, Ala^404^, Thr^302^, Gly^301^, Ala^400^, Lys^603^, Val^399^, Leu^484^, Leu^601^, Ser^349^, Gly^350^	–

a Calculated from docked pose by using the ligand contact tool of *Patch Dock*.

b Hydrogen bond length calculated from docked pose by using *Ligand contact* tool of *Patch Dock*.

### 3.4 Docking of GlcN-6-P synthase with ligand (5a-5d)

GlcN-6-P synthase serves as a target enzyme essential for the biosynthesis of various precursors involved in the formation of the cell wall in both bacteria and fungi [31,64]. Population of bacteria could be stopped by inhibition of this enzyme by a target molecule [65].

**Fig. 6:**
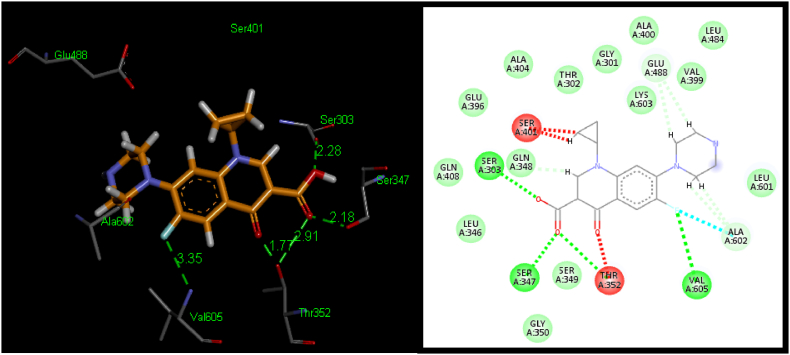
3D (Left) and 2D (Right) binding interactions of standard Ciprofloxacin.

Standard Ciprofloxacin has shown score 4424 and an ACE value −227.85 kcal/mol. Five hydrogen bonds were observed among hotspot and standard Ciprofloxacin. Hydrogen bonding was observed between the hydroxyl group of Ser^303^ and carboxylic acid group of standard Ciprofloxacin. The distance between these bonds is 2.28 Å. Another hydrogen bond was noticed between hydroxyl group of Ser^347^ and carboxylic acid group of Ciprofloxacin (2.18 Å). Third and fourth hydrogen bonds were showed between hydroxy group of Thr^352^ and two carbonyl oxygens of standard Ciprofloxacin with average distance of 1.77 and 2.91 Å. Fifth hydrogen bond was observed between amino group of Val^605^ and fluoro group of Ciprofloxacin (3.35 Å). Hydrophobic interactions of chalcones (**5a-5d**) with Gln^408^, Gln^348^, Leu^346^, Glu^396^, Ala^404^, Thr^302^, Gly^301^, Ala^400^, Lys^603^, Val^399^, Leu^484^, Leu^601^, Ser^349^ and Gly^350^ were also observed (Table 2; Fig. 6).

**Fig. 7:**
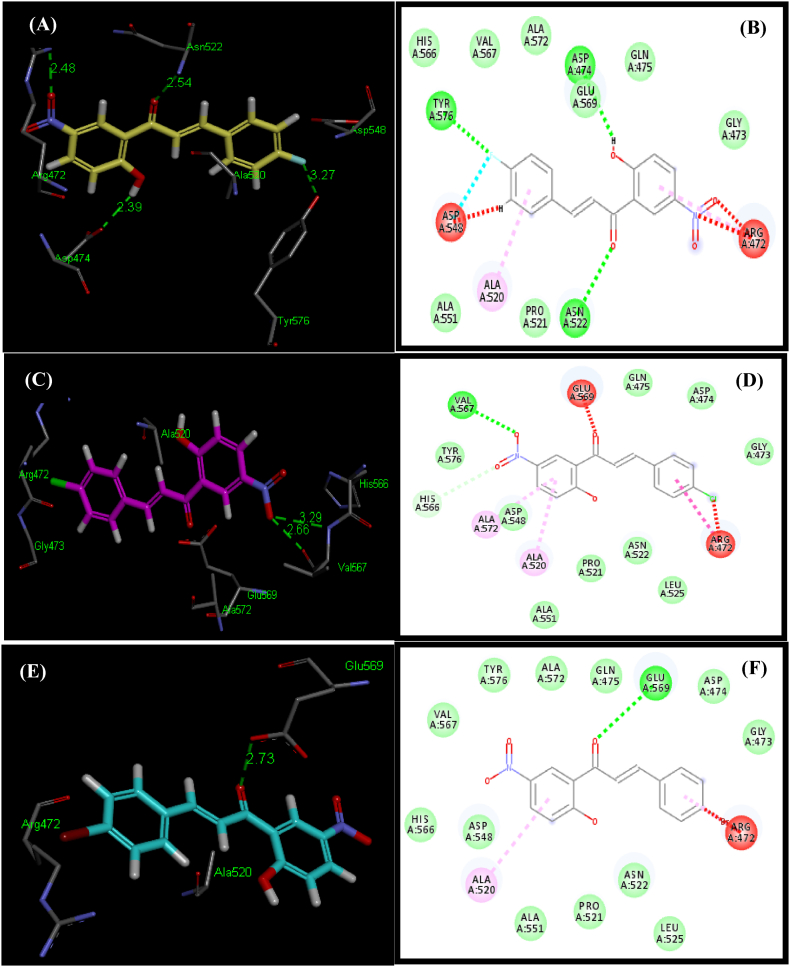

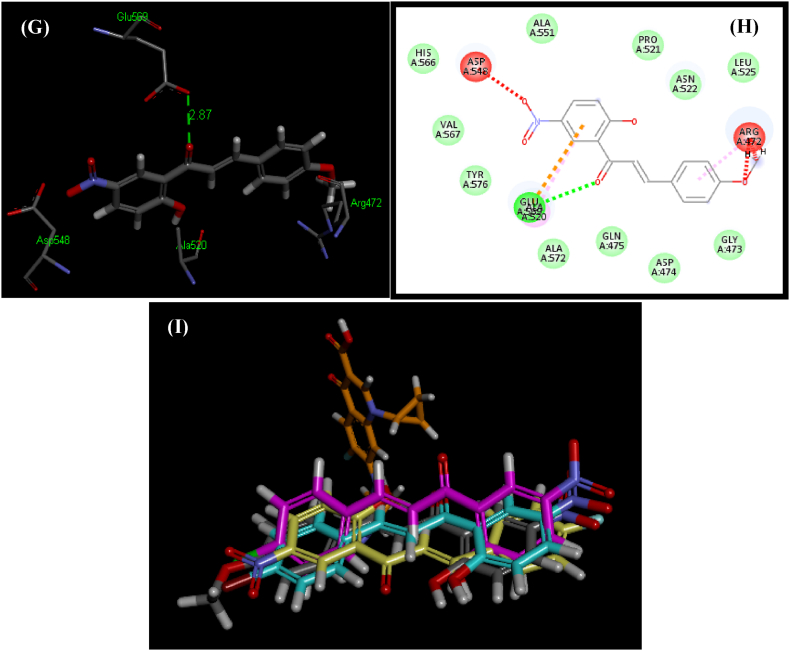
3D and 2D conformations of docked ligands **5a** (A and B), **5b** (C and D), **5c** (E and F), **5d** (G and H) showing interactions within a binding pocket of the GlcN-6-P synthase (PDB ID: 1MOQ). (I) Overlap of bound conformations of ligands **5a** (yellow), **5b** (magenta), **5c** (blue), **5d** (grey) and Ciprofloxacin (mustard).

**Fig. 8:**
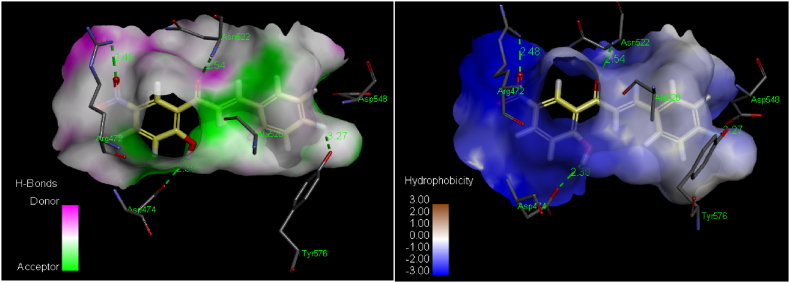
Analyses of hydrogen bonds (left) and hydrophobic contacts (right) of ligand **5a**. Blue color in the hydrophobic interactions identifies favorable structural elements (atoms and torsions) contributing to entire binding energy within the GlcN-6-P synthase (PDB ID: 1MOQ), pink one is unfavorable, and white one is neutral.

Preliminary evaluations of the docked complexes (**5a-5d**) revealed that nearly all ligands showed overlapping with phenyl rings and positioned within the binding pocket (Fig. 7I). Conformations of ligands (**5a-5d**) with high binding affinity for the GlcN-6-P synthase are presented in Fig. 7, Fig. 8.

Table 2 shows that ligand 5a, which had the highest inhibition, also had the highest score (4238; ACE = −160.89 kcal/mol) among the docked complexes. This indicates a strong correlation between experimental and theoretical findings. The docked complex of ligand **5a** exhibited four hydrogen bonds. One hydrogen bond has shown between amino group of Arg^472^ and oxygen of nitro group directly attached with phenyl ring of ligand **5a** (2.48 Å). Hydrogen bonding was noticed between hydroxyl group directly attached with phenyl ring ofTyr^576^ and fluoro group directly attached with phenyl ring of ligand **5a** (3.27 Å). Third hydrogen bond has shown between oxygen of carbonyl group of Asp^474^ and hydroxyl group directly attached with phenyl ring of ligand **5a** (2.39 Å). There is an amino group of Asn^522^ and oxygen of carbonyl group of ligands **5a** potentially forms a hydrogen bond with an average distance of 2.54 Å (Fig. 7A and B). These hydrogen bonds are crucial for the interaction between the ligand and GlcN-6-P synthase. Amino acids His^566^, Val^567^, Ala^572^, Glu^569^, Gln^475^, Gly^473^, Pro^521^ and Ala^551^ showed hydrophobic interactions with ligands (Fig. 8). Pi-alkyl interactions have been visualized between ligand **5a** and Ala^520^, Arg^472^ pocket amino acid residues.

Ligand **5b** exhibited a score (3790; ACE = −92.94 kcal/mol). Two hydrogen bonds were observed; one with NH and other with oxygen of carbonyl group of Val^567^ and hydroxyl of carboxylic acid group of ligand **5b** with average distances of 2.66 and 3.29 Å respectively. The terminal phenyl ring establishes hydrophobic connections with the hydrophobic side chains of key amino acids, including Glu475, Asp474, Gly473, Leu525, Asn522, Pro521, Ala551, Asp548, and Tyr576, within van der Waals interaction ranges (Table 2; Fig. 7C and D). Pi-alkyl interactions have been visualized among ligand **5b** and Ala^520^, Ala^572^ binding pocket amino acid residues.

As showed in Table 2; ligand **5c** with the score (3896; ACE = – 92.78 kcal/mol) showed a strong similarity between experimental and theoretical studies. Docked complex of ligand **5c** showed hydrogen bonding between Glu^569^ and carbonyl group of ligand **5c** (2.73 Å). This hydrogen bond is regarded as to play a crucial role in the interaction of the ligand with the GlcN-6-P synthase (Fig. 7E and F). There were numerous hydrophobic interactions of the ligand with the binding pocket amino acids residues Val^567^, Tyr^576^, Ala^572^, Gln^475^, Asp^474^, Gly^473^, Leu^525^, Asn^522^, Pro^521^, Ala^551^, Asp^548^ and His^566^ observed. Pi-alkyl interactions have been showed between ligand **5c** and Ala^520^, Arg^472^ pocket amino acid residues.

Ligand **5d** illustrated a high score (3958; ACE = −102.77 kcal/mol). Evaluation of the interactions with the GlcN-6-P synthase binding pocket depicted hydrogen bond as well as hydrophobic interactions (Fig. 7G and H). A hydrogen bond has shown between the hydrogen of hydroxyl group of Glu^569^ and oxygen of carbonyl group attached to the ligand **5d** (2.87 Å). The terminal phenyl ring establishes hydrophobic connections with the hydrophobic side chains of key amino acids including Ala^551^, Pro^521^, Asn^522^, Leu^525^, Gly^473^, Asp^474^, Gln^475^, Ala^572^, Tyr^576^, Val^567^ and His^566^ residues within van der Waals interaction ranges. Pi-alkyl interaction has shown between ligand **5d** and Ala^520^, Arg^472^ hotspot amino acid.

However, ligand **5a** exhibited higher score compared to other ligands which correspond with the antibacterial screening outcomes. The critical amino acids exhibited in the hotspot are Glu^475^, Asp^474^, Gly^473^, Leu^525^, Asn^522^, Pro^521^, Tyr^576^. This computational study closely aligns with the findings from antibacterial screening, affirming the significant impact of the molecular docking analysis.

## 4 Conclusions

A series of Chalcones (**5a-5d**) have been successfully synthesized by Claisen-Schmidt condensation reaction. Among all the synthesized compounds, the chalcones (**5a** and **5d**) exhibited excellent antimicrobial activity as compare to reference drug and other synthesis compounds. The chalcones **5c** was proved to be less effective. Antibiofilm activity of (**5a-5d**) were tested against pathogens (*E. coli, P. aeuroginosa, K. pneumoniae and S. aureus*). The compound **5a**, **5b** and **5d** showed excellent inhibitory efficacy while the compound **5a** and **5d** showed significant antibiofilm activity against all four pathogens as compared to reference Ciprofloxacin. All the chalcones (**5a-5d**) were also docked using *PachDock* molecular docking software and exhibited that ligand **5a** showed excellent results with score 4238 and ACE value −160.89 kcal/mol which is in concordance with the antibacterial activity outcomes. Results of Chalcones formulations as bacterial growth inhibition and as antibiofilm agents seems promising and open new avenues towards drug discovery, however, further investigations are still needed. Future implications of the present study will be helpful for the success of health care systems besides overcoming the issues of emerging drug resistance in pathogens.

## Data availability statement

The original contributions presented in the study are included in the article, further inquiries can be directed to the corresponding author/s. Data will be made available on request.

## CRediT authorship contribution statement

**Tariq Nawaz:** Writing – original draft, Software, Resources, Project administration, Methodology, Investigation. **Affifa Tajammal:** Writing – review & editing, Visualization, Validation, Supervision, Software, Conceptualization. **Aisha Waheed Qurashi:** Validation, Supervision, Software, Resources, Methodology. **Mehr-un Nisa:** Writing – review & editing, Validation, Software, Resources. **Dalal Nasser Binjawhar:** Writing – review & editing, Validation, Software, Resources, Funding acquisition. **Munawar Iqbal:** Writing – review & editing, Validation, Resources.

## Declaration of competing interest

The authors declare no conflict of interests.
