# Supplementary material for: Synthesis, antibacterial, antibiofilm, and docking studies of chalcones against multidrug resistance pathogens
Source: Heliyon. 2024 May 6;10(13):e30618. doi: 10.1016/j.heliyon.2024.e30618 (PMC11263648; doi:10.1016/j.heliyon.2024.e30618)
Supplement: Multimedia component 1 [file mmc1.docx]

Appendix A

**Supplementary Data**

Tariq Nawaz^1^, Affifa Tajammal^1^*, Aisha Waheed Qurashi^2^, Mehr-un-Nisa^3^, , Dalal Nasser Binjawhar^3,4^ and Munawar Iqbal^3^

^1^Department of Chemistry, Lahore Garrison University, Pakistan

^2^Department of Biology, Lahore Garrison University, Pakistan

^3^Department of Chemistry, University of Lahore, 1-KM Defense Road, Lahore, Pakistan

^4^Department of Chemistry, College of Science, Princess Nourah bint Abdulrahman University, P.O. Box 84428, Riyadh 11671, Saudi Arabia

* Corresponding author

Corresponding author mailing address:

[Affifa.tajammal@lgu.edu.pk](mailto:Affifa.tajammal@lgu.edu.pk), Affifa.tajammal@yahoo.com


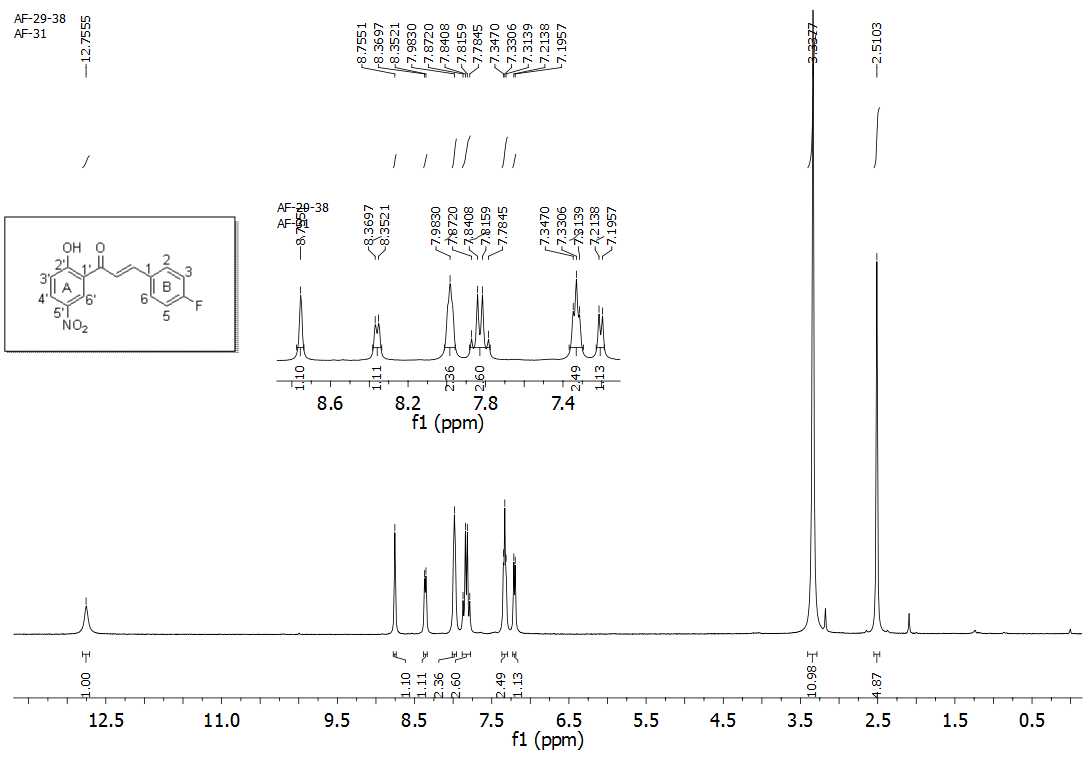


**Fig 1: ^1^H NMR of 5a**


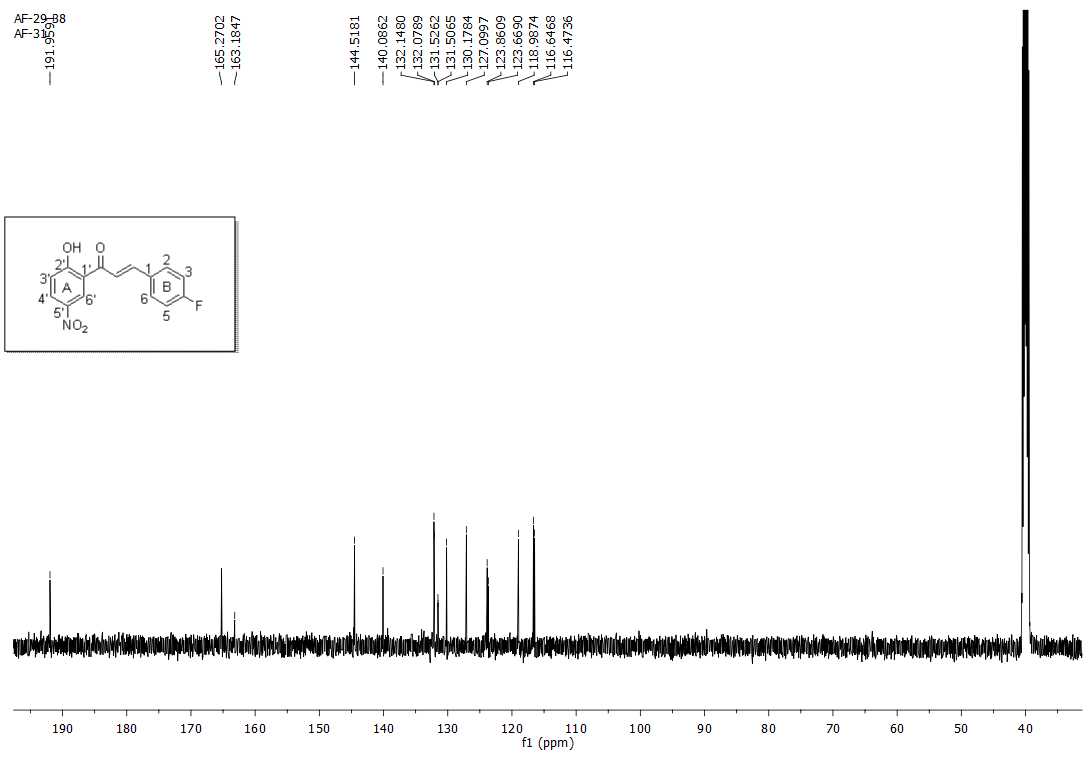


**Fig 2: ^13^C NMR of 5a**


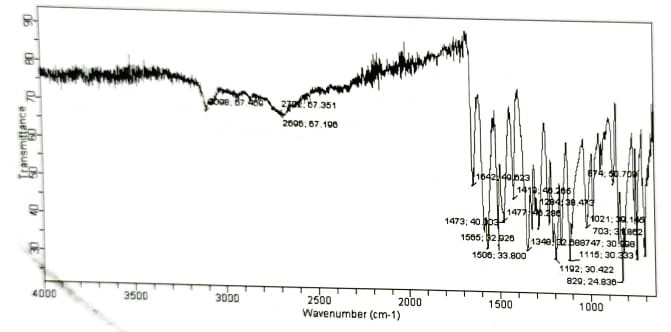


**Fig 3: FTIR spectra of compound 5a**


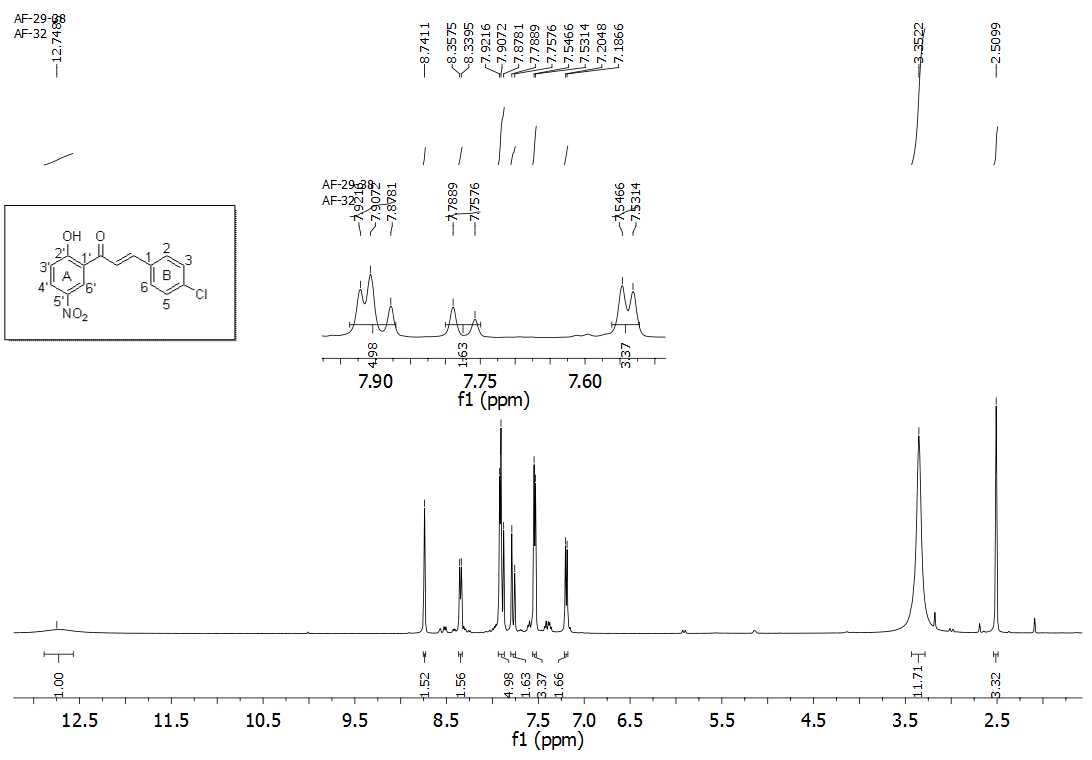


**Fig 4: ^1^H NMR of 5b**


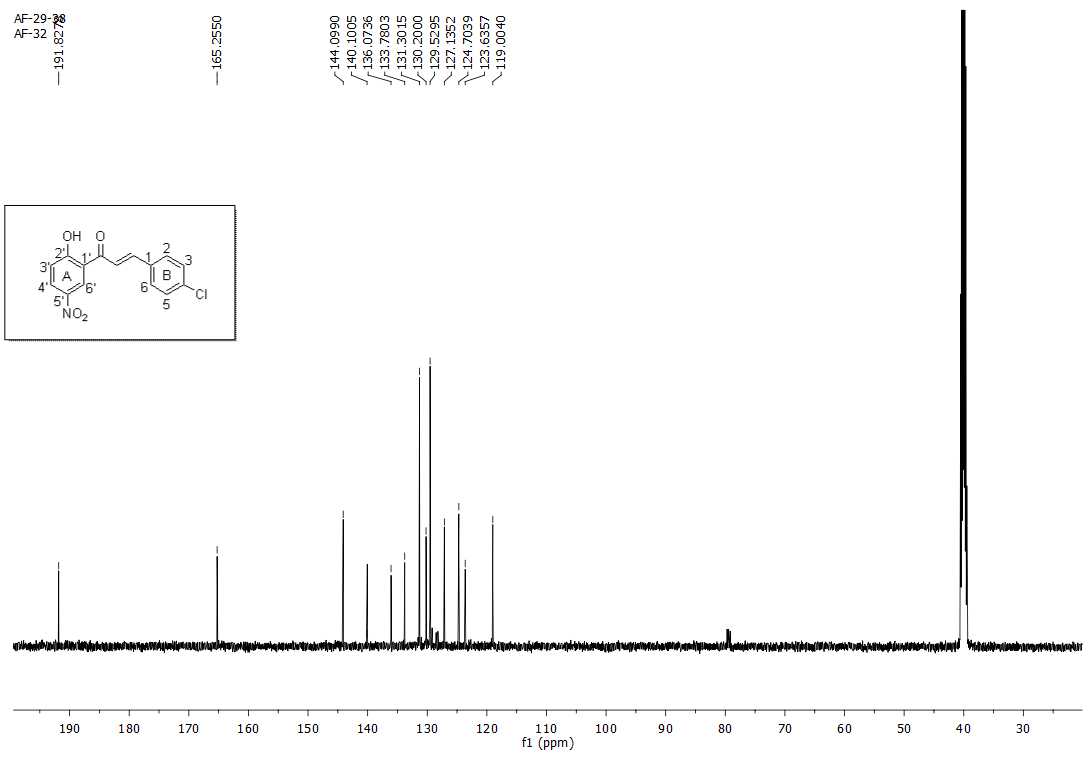


**Fig 5: ^13^C NMR of 5b**


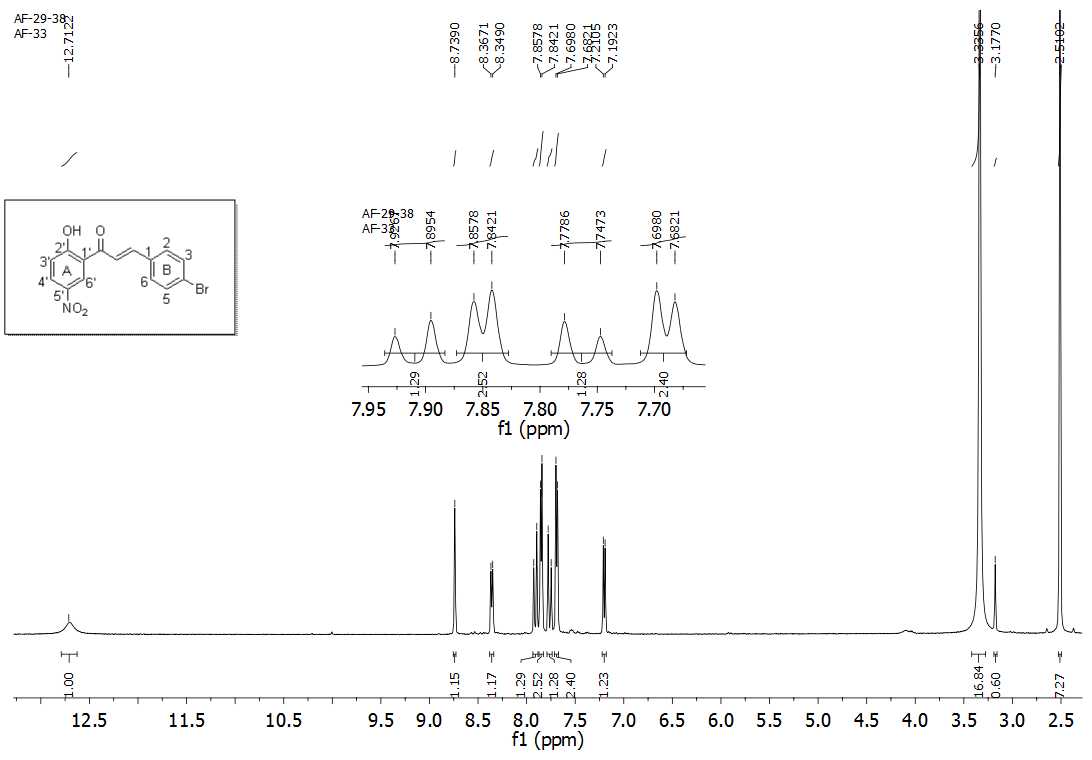


**Fig 6: ^1^H NMR of 5c**


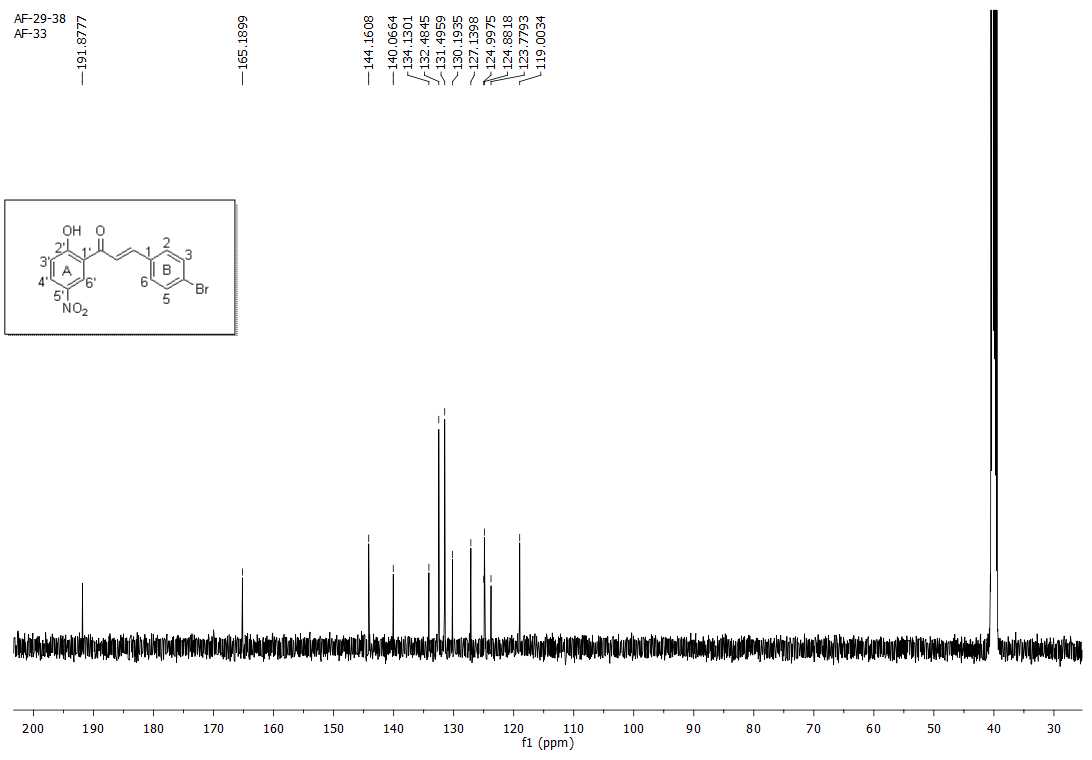


**Fig 7: ^13^C NMR of 5c**


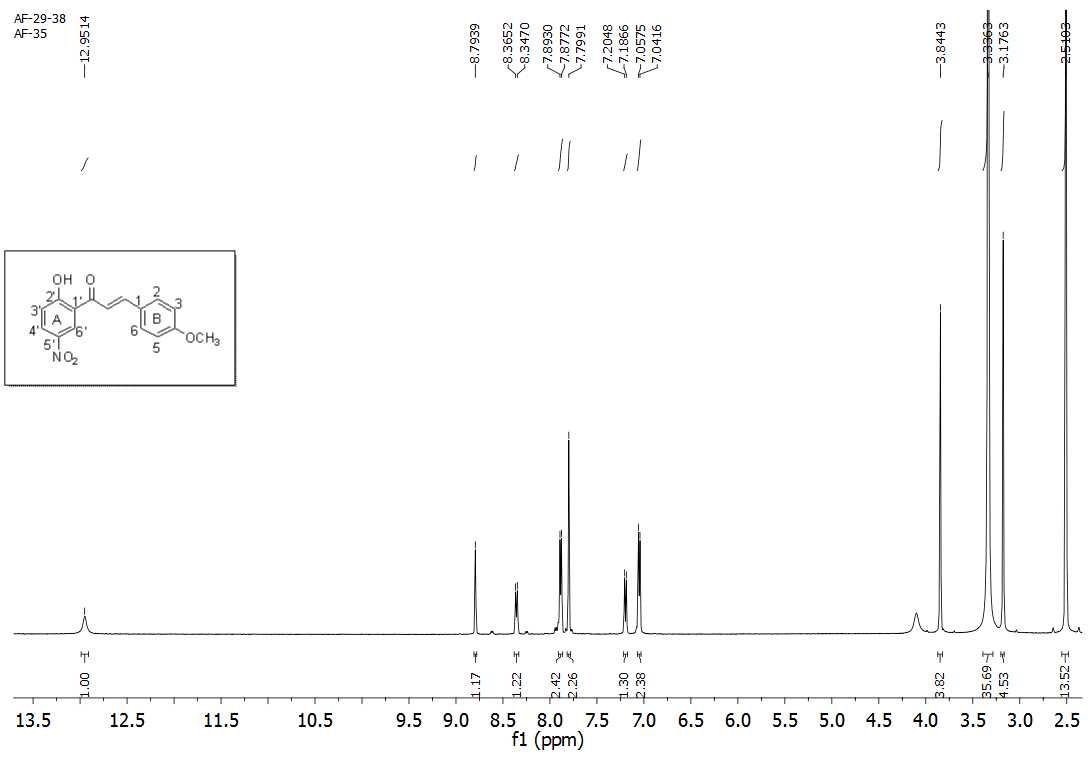


**Fig 8: ^1^H NMR of 5d**


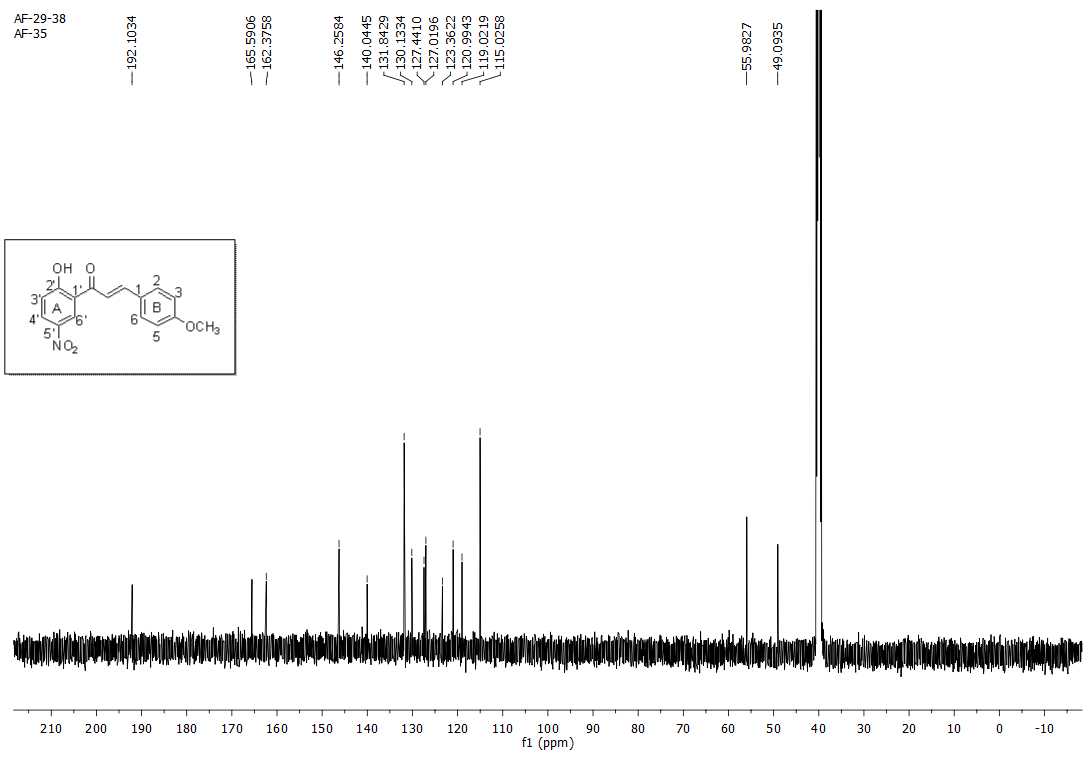


**Fig 9: ^13^C NMR of 5d**


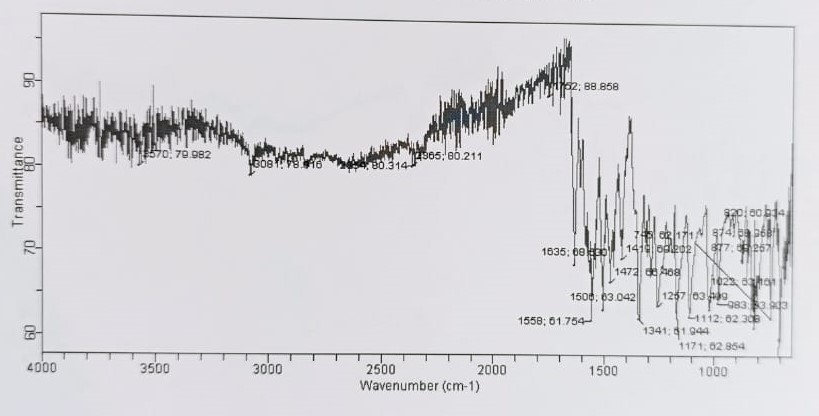


**Fig 10: FTIR spectra of compound 5d**
